# Trapping intermediate MLCT states in low-symmetry {Ru(bpy)} complexes†
†Electronic supplementary information (ESI) available. See DOI: 10.1039/c7sc02670f



**DOI:** 10.1039/c7sc02670f

**Published:** 2017-08-29

**Authors:** Alejandro Cadranel, Paola S. Oviedo, German E. Pieslinger, Shiori Yamazaki, Valeria D. Kleiman, Luis M. Baraldo, Dirk M. Guldi

**Affiliations:** a Department of Chemistry and Pharmacy , Interdisciplinary Center for Molecular Materials (ICMM) , Friedrich-Alexander-Universität Erlangen-Nürnberg , Egerlandstr. 3 , 91058 Erlangen , Germany . Email: ale.cadranel@fau.de ; Email: dirk.guldi@fau.de; b Departamento de Química Analítica , Inorgánica y Química Física , INQUIMAE , Facultad de Ciencias Exactas y Naturales , Universidad de Buenos Aires , Ciudad Universitaria , Pabellón 2 , C1428EHA , Buenos Aires , Argentina; c Department of Chemistry , University of Florida , PO BOX 117200 , Gainesville , FL 32611-7200 , USA

## Abstract

The picosecond excited state dynamics of [Ru(tpm)(bpy)(NCS)]^+^ (**RubNCS^+^**) and [Ru(tpm)(bpy)(CN)]^+^ (**RubCN^+^**) (tpm = tris(1-pyrazolyl)methane, bpy = 2,2′-bipyridine) have been analyzed by means of transient absorption measurements and spectroelectrochemistry. Emissive ^3^MLCTs with (GS)HOMO(h^+^)–(GS)LUMO(e^–^) configurations are the lowest triplet excited states regardless of whether 387 or 505 nm photoexcitation is used. 387 nm photoexcitation yields, after a few picoseconds, the emissive ^3^MLCTs. In contrast, 505 nm photoexcitation populates an intermediate excited state that we assign as a ^3^MLCT state, in which the hole sits in a metal-centered orbital of different symmetry, prior to its conversion to the emissive ^3^MLCTs. The disparities in terms of electronic configuration between the intermediate and the emissive ^3^MLCTs have two important consequences. On one hand, both states feature very different fingerprint absorptions in transient absorption measurements. On the other hand, the reconfiguration is impeded by a kinetic barrier. As such, the conversion is followed spectroscopically and kinetically on the 300 ps timescale.

## Abstract 2

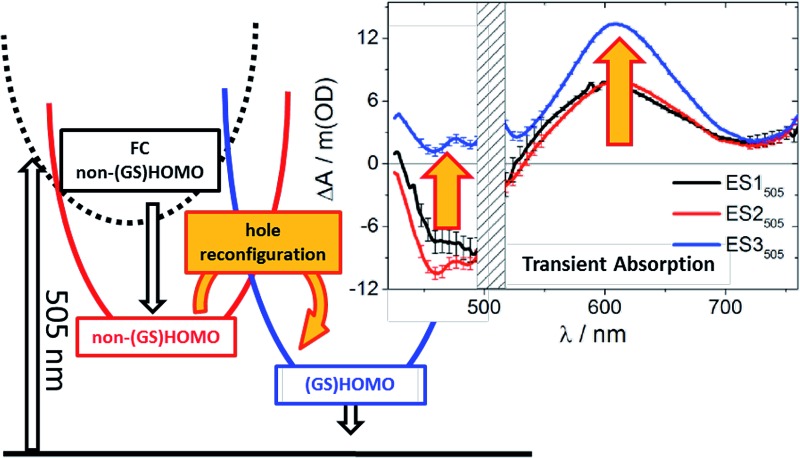
The picosecond excited state dynamics of [Ru(tpm)(bpy)(NCS)]^+^ (**RubNCS^+^**) and [Ru(tpm)(bpy)(CN)]^+^ (**RubCN^+^**) (tpm = tris(1-pyrazolyl)methane, bpy = 2,2′-bipyridine) reveal an intermediate MLCT excited state living in the 300 ps timescale.

## Introduction

The MLCT excited state manifold of ruthenium polypyridines constitutes a unique playground for extraordinary photophysics and photochemistry. Their high versatility in terms of both structural modifications and ligand substitutions allows for the fine tuning of their excited state properties.^1–4^ Many attempts have been made to exploit them as integrative components of supramolecular architectures for catalysis and energy conversion.^5–13^ MLCT manifolds are typically populated upon visible light absorption. Such MLCT excited states involve a hole in the parent octahedral t_2g_ ruthenium orbitals and an excited electron in a π* orbital of the polypyridinic ligand. One of the greatest challenges in the field is to gain full control over these excited states. If successful, this would assist in promoting charge separation and ultimately using these redox equivalents to catalyze specific reactions or to collect them at electrodes.

In a general scenario, the guiding of the excited state energy or charges requires unidirectional energy and/or electron transfer processes. Depending on the precise reaction mechanism, a myriad of energy or symmetry requirements will need optimization. One of the more conventional approaches relies on tuning the energetics of heteroleptic or mixed-ligand complexes. Upon populating the Franck–Condon excited states, differences in the reduction potentials between the non-equivalent ligands promote efficient inter ligand charge transfer (ILCT) events on multiple timescales.^14^ It is implicit that an electron potentially hops from one ligand to another and, in turn, is directed to the energetically most favorable orbital.^15–19^ Once the electron reaches the energetically lowest orbital it becomes accessible as a reductive equivalent for catalysis or for injection into electrodes.^20^

We envision an alternative strategy based on the symmetry of MLCT excited states, rather than on their energetics, and a judicious choice of ancillary ligands. The excited metal ion features three t_2g_ orbitals on which the hole usually sits. An unsymmetrical coordination splits these orbitals in energy affording energy or electron donors of vastly different symmetry. To the best of our knowledge, the only evidence for MLCT states of different symmetry has been documented for [Os(phen)_3_]^2+^.^21^ In this complex, the lowest MLCT state presents a transient absorption band in the near-infrared region which was ascribed to an interconfigurational dπ → dπ transition. This photoinduced absorption transition results in a MLCT state of different symmetry than the lowest one. However, it has never been directly observed. The lack of clear-cut cases demonstrates the need for a better understanding of the factors that determine the population of MLCT states of different symmetry.

**Fig. 1:**
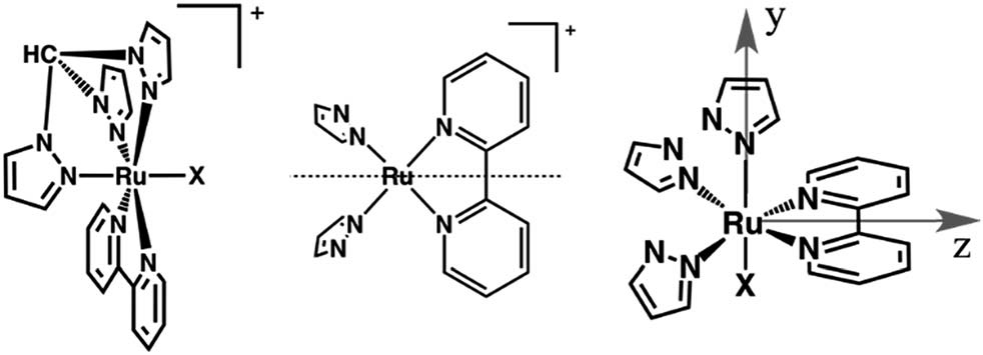
Left: Sketches of the [Ru(tpm)(bpy)X]^+^ complexes studied in this work, **RubNCS^+^** (X = NCS^–^) and **RubCN^+^** (X = CN^–^). Center: The dotted line indicates the reflection plane that bisects the bpy ligand, determining the C_s_ symmetry. Right: Axis denomination for **RubNCS^+^** and **RubCN^+^**. The *x* axis is normal to the page.

In this study, we establish the means by which light absorption in a particular region of the visible spectrum results in the population of an intermediate excited state, that we assign as a high energy ^3^MLCT state. There, the hole is likely to sit in a metal-based orbital of different symmetry than those in the four lowest-lying ^3^MLCT states described by Kober and Meyer.^22^ We show for the first time that high-energy {Ru(bpy)} MLCT excited states are trappable, potentially allowing for the utilization of their energy before dissipation. To shed light on this phenomenon we take advantage of the synergistic combination of transient absorption and spectroelectrochemistry,^23,24^ investigating the ultrafast dynamics of mixed-ligand ruthenium polypyridines. [Ru(tpm)(bpy)(NCS)]^+^ (**RubNCS^+^**) and [Ru(tpm)(bpy)(CN)]^+^ (**RubCN^+^**) were selected as models because of their overall low symmetry (C_s_ point group), and because they contain ligands with very different donor properties, which should result in a split of the energy of the dπ orbitals of the ruthenium ions (Fig. 1). The selected ligands are prone to participate in LMCT transitions and hence could provide information on the hole dynamics in these compounds.

## Results

### Spectroelectrochemistry

**Fig. 2:**
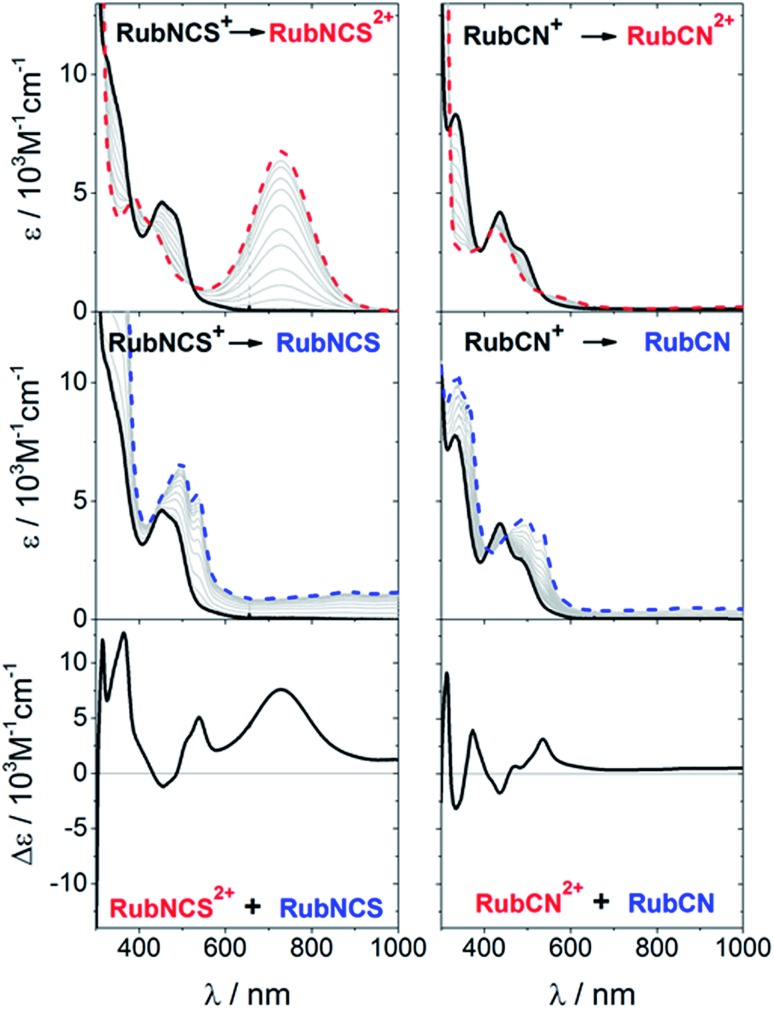
Upper panel: Spectral evolution during the Ru(ii) → Ru(iii) oxidation of **RubNCS^+^** and **RubCN^+^** in acetonitrile (0.1 M [TBA]PF_6_); initial: solid black line, final: dashed red line, intermediate: grey lines. Middle panel: Spectral evolution during the one-electron reduction of **RubNCS^+^** and **RubCN^+^** in acetonitrile (0.1 M [TBA]PF_6_); initial: solid black line, final: dashed blue line, intermediate: grey lines. Bottom panel: Sum of the oxidative and reductive differential spectra of **RubNCS^+^** and **RubCN^+^** in acetonitrile.

The UV-vis absorption spectra of **RubNCS^+^** and **RubCN^+^** were previously reported.^25^ Fig. 2 shows the spectroelectrochemical evolution throughout the visible region upon one electron oxidation and one electron reduction. As the oxidations are metal-centered,^25^ the electrolysis of **RubNCS^+^** and **RubCN^+^** at anodic potentials produces a decrease in the MLCT absorptions due to Ru(ii) depletion. In the oxidized forms, the absorption features peaks at 391 nm (**RubNCS^2+^**) and 425 nm (**RubCN^2+^**), which are assigned to LMCT dπ(Ru) ← π(heterocycle) transitions.^26–29^ A strong LMCT transition is observed at 730 nm for **RubNCS^2+^**. Similar fingerprints are observed for related complexes^23,30–32^ and, in turn, we ascribe them to dπ(Ru) ← π(NCS) charge transfer transitions. **RubCN^2+^** shows weaker transitions at 590 nm, which are also assigned to LMCT transitions. These are likely dπ(Ru) ← π(tpm), since tpm is a stronger electron donor than bpy and CN^–^.

As previously reported,^25^ the first reduction of **RubNCS^+^** and **RubCN^+^** is bpy-centered. This is simply understood in terms of the more extended conjugation found in bpy relative to tpm. Support for this assumption is based on the spectral changes for **RubNCS** and **RubCN**, which are in sound agreement with those seen for the reduction of [Ru(bpy)_3_]^2+^.^33^ In the reduced forms **RubNCS** and **RubCN**, the absorptions in red are ascribed to π*(heterocycle) ← π*(radical anion) transitions.‡
‡The cyclic voltammetric measurements of saturated solutions of tpm in acetonitrile failed to disclose any appreciable cathodic processes all the way up to –2.5 V *vs.* Ag/AgCl (Fig. S1†), hampering the recording of spectral fingerprints associated with the tpm reduction.


Spectroelectrochemistry has been successfully used to assign the patterns observed in the differential spectra of the MLCT excited states of ruthenium polypyridines.^24,34–40^ Fig. 2 shows the differential changes for metal oxidation (upper panel) and ligand reduction (middle panel) and both contributions summed up (bottom panel) for **RubNCS^+^** and **RubCN^+^**. The weak intensity of the negative bands, in the regions where the MLCT bands are located, should be noted. This is due to a LMCT feature in the oxidized form, which compensates for the MLCT bleaching.

### Transient absorption experiments

**Fig. 3:**
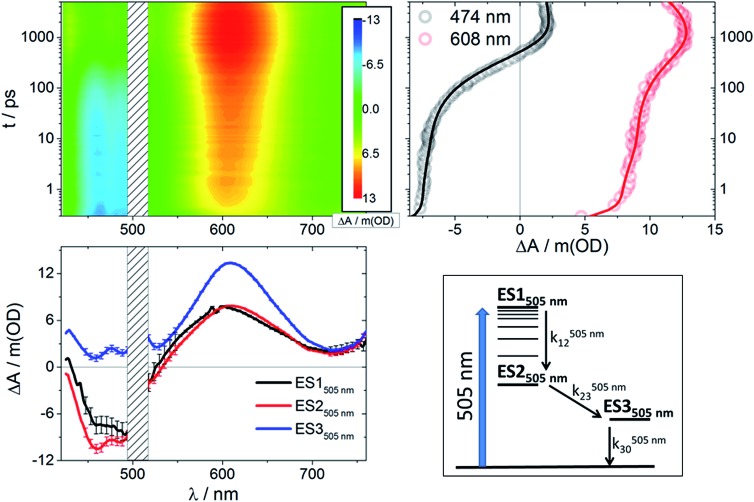
Upper left: Differential absorption 3D map obtained from femtosecond pump-probe experiments (*λ*_ex_ = 505 nm) on **RubNCS^+^** in DMSO at room temperature. Upper right: Time absorption profiles (open circles) and corresponding fittings from target analysis (solid lines) for **RubNCS^+^** upon excitation at 505 nm using the model presented in the bottom right side of this figure. Bottom left: Species-associated differential spectra for **RubNCS^+^** upon excitation at 505 nm; ES1_505 nm_: black, ES2_505 nm_: red, ES3_505 nm_: blue. Bottom right: Target model proposed to fit the data.

Femtosecond transient absorption measurements were performed for **RubNCS^+^** and **RubCN^+^** in argon-deoxygenated DMSO solutions at room temperature. The upper panels of Fig. 3 and S2† show the broadband differential absorption spectral maps and kinetic traces at selected wavelengths upon 505 nm (2.46 eV) illumination. Under these conditions, both complexes exhibit bleaching in the 450–500 nm range at short time delays. Remarkably, within hundreds of ps they transform into photoinduced absorptions (PIAs) with maxima at 475 and ≈508 nm (**RubNCS^+^**)§
§The last maximum is obscured by pump scattering. and 475 nm (**RubCN^+^**).

Notably, the presence of a NCS^–^ ligand induces an additional LMCT transition in the differential absorption spectra of **RubNCS^+^** with a maximum at 600–610 nm. Its temporal evolution is in the time range of hundreds of ps. In comparison to the spectroelectrochemical features (Fig. 2), the observed LMCT transitions are blue-shifted due to metal orbital destabilization by the imine radical anion of the MLCT excited state. The cyanide complex gives rise to weaker photoinduced absorptions owing to the fact that in this case, only the dπ(Ru) ← π(heterocycle) LMCT and π*(heterocycle) ← π*(radical anion) transitions contribute in this spectral region.

**Fig. 4:**
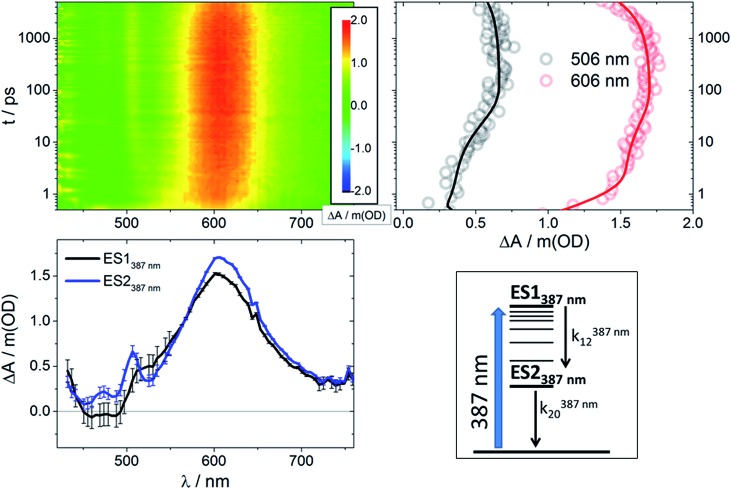
Upper left: Differential absorption 3D map obtained from femtosecond pump-probe experiments (*λ*_ex_ = 387 nm) on **RubNCS^+^** in DMSO at room temperature. Upper right: Time absorption profiles (open circles) and corresponding fittings from target analysis (solid lines) for **RubNCS^+^** upon excitation at 387 nm using the model presented at the bottom right side of this figure. Bottom left: Species-associated differential spectra for **RubNCS^+^** upon excitation at 387 nm; ES1_387 nm_: black, ES2_387 nm_: blue. Bottom right: Target model proposed to fit the data.

Interestingly, the differential transient absorption signals observed upon 387 nm (3.20 eV) irradiation are strikingly different (Fig. 4 and S3†). **RubNCS^+^** and **RubCN^+^** lack any bleaching. For **RubCN^+^**, the only signal below 550 nm arises from PIA at 475 nm, corresponding to the LMCT transitions. **RubNCS^+^** shows similar transitions at 473 and 506 nm, together with a LMCT PIA at 600–610 nm. These bands are remarkably similar to those observed upon irradiation at 505 nm after 1 ns (Fig. S6 and S8†).

Complementary nanosecond measurements with 505 nm (Fig. S6 and S8†) or 387 nm (Fig. S7 and S9†) pumps point to the presence of a single excited state in the long timescale. These nanosecond differential spectra and their corresponding lifetimes (Table S1†) fully match the spectra observed at the end of the picosecond experiments (Fig. S10†).

### Kinetic analysis of the transient experiments and decay model

Global fitting of the data after 505 nm excitation led to three different transient species in each case (Fig S4†): a short-lived component with *τ* < 6 ps, an intermediate-lived component with *τ* on the order of hundreds of picoseconds, and a long-lived component that decays on the nanosecond timescale. With the aforementioned data in hand, we performed target analyses^38,41^ based on the model depicted in Fig. 3 (bottom right). The resulting species-associated differential spectra and time constants are presented in Fig. 3 (bottom left) and Table 1, respectively. The validity of the model is discussed below.

**Table 1:** Time constants extracted from picosecond TA experiments using the models depicted in Fig. 3 and 4

X	*λ*_pump_ = 387 nm		*λ*_pump_ = 505 nm		
	*k*_12_/ps^–1^ (*τ*_12_/ps)	*k*_20_/ns^–1^ (*τ*_20_/ns)	*k*_12_/ps^–1^ (*τ*_12_/ps)	*k*_23_ × 10^3^/ps^–1^ (*τ*_23_/ps)	*k*_30_/ns^–1^ (*τ*_30_/ns)
**NCS^–^**	0.262 ± 0.002 (3.8)	0.028 ± 0.001 (35.9)	0.330 ± 0.006 (3.0)	2.67 ± 0.01 (375)	0.032 ± 0.001 (31.1)
**CN^–^**	0.41 ± 0.01 (2.4)	nd (106)^*a*^	0.54 ± 0.02 (1.9)	3.34 ± 0.05 (300)	nd (112.5)^*a*^

^*a*^Values extracted from nanosecond TA experiments. nd: non determined in picosecond experiments.

Following excitation at 505 nm, both compounds give rise to an initial excited state spectrum (ES1_505 nm_) typical of the ^3^MLCT states seen in ruthenium polypyridine complexes,^24,37,40,42–45^ with bleaching in the spectral area of the ground state absorption and a weaker positive transient in the spectral range of >550 nm (3D maps and ES1_505 nm_ in Fig. 3 and S2†). For the thiocyanate complex an intense dπ(Ru) ← π(NCS) LMCT transition was found around 600–610 nm.^23^ The second species (ES2_505 nm_) has a very similar spectral pattern. Both complexes reveal a third component (ES3_505 nm_) with a distinct additional photoinduced absorption at <550 nm, but no bleaching was observed in this spectral region. Also, for the thiocyanate complex, an enhancement of the LMCT (600–610 nm) band relative to the short timescale species is clearly discernable.

Global analysis of the results following photoexcitation at 387 nm leads to only two different transient species (Fig. S5†): a short-lived component with *τ* < 6 ps and a long-lived component with *τ* on the nanosecond timescale. Target analyses employed with the two-excited-state model depicted in Fig. 4 (bottom right) result in the spectral data presented in Fig. 4 (bottom left) with the time constants listed in Table 1. Immediately upon 387 nm photoexcitation, **RubNCS^+^** and **RubCN^+^** show a lack of ground state bleaching (3D maps and ES1_387 nm_ in Fig. 4 and S3†). The initial species (ES1_387 nm_) transforms into the final form (ES2_387 nm_) very rapidly and without significant modifications to the spectrum. **RubNCS^+^** shows the presence of strong LMCT photoinduced absorptions at longer wavelengths. Its spectrum looks strikingly similar to that recorded at long time delays following 505 nm photoexcitation (ES3_505 nm_).

**Fig. 5:**
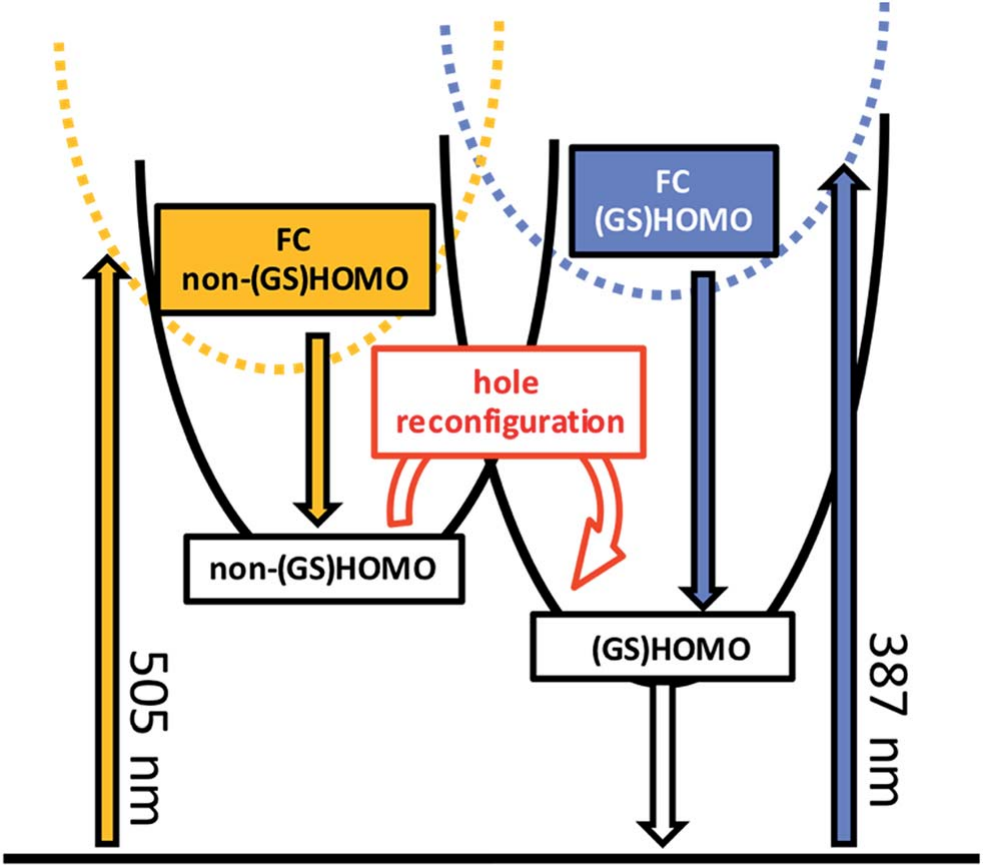
Proposed model describing the photophysical behavior observed for **RubNCS^+^** and **RubCN^+^** upon 505 nm (orange) or 387 nm (blue) illumination. Labels indicate the hole configuration. Highlighted is the proposed hole reconfiguration process characterized by *k*_23_.

Prior to addressing the nature of the different excited states, a few remarks on the target models are needed. Given the different lifetimes obtained for the two components found following UV (387 nm) photoexcitation, only a sequential model seems applicable (Fig. 5). Upon visible (505 nm) photoexcitation, three components were identified and two potential models were considered. A branched model, in which ES1_505 nm_ feeds ES2_505 nm_ and ES3_505 nm_, both of which depopulate independently *via* ground state (GS) recovery, does not give satisfactory results. It results in an ES3_505 nm_ spectrum with very broad negative signals at wavelengths longer than 550 nm. In the experimental spectra neither stimulated emission nor GS absorption is noted at long wavelengths. Consequently, this branched model fails to reflect the fast dynamics accurately. A second model, with sequential steps, was applied, in which ES1_505 nm_ feeds ES2_505 nm_ and subsequently ES3_505 nm_. This sequential model yields three individual spectra that describe the experimental findings very well (Fig. 3).

Regardless of the excitation wavelength, similar long-time dynamics were observed and confirmed by independent picosecond and nanosecond measurements, yielding identical spectra (Fig. S10†) and time constants (Tables 1 and S1†). In line with the previous reports^25^ the nanosecond measurements show quantitative GS recovery. Upon photoexcitation in different regions of the spectra, both deactivation cascades proceed through different mechanisms, leading to a common excited state with the corresponding lifetimes matching the emission lifetimes.^25^

## Discussion

At the 387 nm and 505 nm excitation wavelengths, which relate to 3.20 eV/25 800 cm^–1^ and 2.46 eV/19 800 cm^–1^, respectively, the GS absorptions of **RubNCS^+^** and **RubCN^+^** are largely dominated by ^1^MLCT transitions.^25^ On the femtosecond timescale, intersystem crossing (ISC) between singlet and triplet manifolds leads to the population of the ^3^MLCT manifold, similar to the results found for other ruthenium polypyridine complexes.^46^ Following the initial ISC, ground-state absorption bleaching is observed in the differential absorption spectra only upon 505 nm photoexcitation. As such, we propose that different triplet excited states are populated on the early timescale when using different excitation wavelengths.

From the good agreement between the transient absorption lifetimes and emission lifetimes on the nanosecond timescale, we conclude that ES2_387 nm_ and ES3_505 nm_ are the same states, namely the emissive ^3^MLCT excited state. The spectral profiles of the emissive states are in good agreement with the spectroelectrochemical assays. Our interpretation of the differential absorption spectra of the long-time emissive states based on the spectroelectrochemical results is therefore appropriate. Thus, we assign a (GS)HOMO(h^+^)–(GS)LUMO(e^–^) electronic configuration to these excited states. This is in line with the notion that these excited states are the lowest ^3^MLCTs.

Considering that the spectral features of ES1_387 nm_ are not markedly different from those of the emissive ^3^MLCT, we ascribe them to a Franck–Condon state with an electronic configuration similar to that of the emissive states. *k*_12_^387 nm^ is assigned to the development of the MLCT manifold^47–51^ and occurs within a few picoseconds.

When the excitation is shifted to lower energies, namely 505 nm, we observed a different behavior, since in both complexes the initial differential absorption spectra are dominated by a negative signal. This contrasts with the spectra observed upon photoexcitation at 387 nm, which fail to exhibit any negative absorptions. This disparate response suggests that the electronic configuration of ES1_505 nm_ differs from that of ES1_387 nm_.

We considered different alternatives for the identity of ES1_505 nm_. In systems featuring π-extended ligands or donor/acceptor groups, ^3^IL (intra-ligand π–π*)^52–57^ or ^3^ILCT (inter-ligand charge transfer)^54,58,59^ states play the role of energy reservoirs. In our case of {(tpm)(bpy)}, it is unlikely to deal with states at energies low enough to equilibrate with or be thermally populated from ^3^MLCTs. Firstly, IL states are not observed in [Ru(bpy)_3_]^2+^.^43,46,47,60^ Secondly, tpm GS π–π* absorptions occur around 200 nm, an energy much higher than our excitation. Thirdly, if ES1_505 nm_ was an ILCT state, a hole shift from the Ru(iii) present in the initially populated MLCT to the tpm would be required. However, tpm and bpy are harder to oxidize than Ru(ii) and, in turn, it is unlikely that holes move in the excited state to any of the iminic ligands, precluding the participation of any ILCT state.

Alternatively, ^3^MC states could account for ES1_505 nm_. ^3^MC states are, however, not observed upon 387 nm photoexcitation, despite the 0.74 eV or 6000 cm^–1^ of excess electronic and vibrational energy in comparison with the 505 nm excitation. This is inconsistent with a thermally activated process as seen, for example, in the ^3^MLCT to ^3^MC state transformation.^61,62^ Furthermore, in the ^3^MC states, Ru(ii) features unpaired electrons in both parent t_2g_ and e_g_ orbitals, enabling dπ(Ru) ← π(NCS) LMCT absorptions. Importantly, such LMCT absorptions, which originate from the ^3^MC states, are blue-shifted with respect to those originating from the ^3^MLCT states, as observed in similar systems.^23^ For ES1_505 nm_, a PIA at 600–610 nm is observed at the same energy and with the same shape as that assigned to a LMCT transition in ES1_387 nm_. Both of these observations point to the MLCT nature of ES1_505 nm_, and, thus, we assign it as a high energy ^3^MLCT.¶
¶In the complexes reported here, depopulation of the long-lived emissive ^3^MLCT state is linked to GS recovery. ^3^MC excited states are, however, likely to be thermally populated from the emissive ^3^MLCT excited state and reduce the overall lifetimes relative to the case of [Ru(bpy)_3_]^2+^. Therefore, depopulation of the emissive states includes direct conversion to the GS and also an intermediate internal conversion step to the ^3^MC excited states, whose lifetimes are too short to be detected.^62^



As ES1_505 nm_ and the emissive ES3_505 nm_ states are both best described as ^3^MLCTs with a bpy-localized orbital occupied by an excited electron and a metal-centered hole, they should bear the excited hole or the excited electron in different orbitals. If the difference between these states is based on the location of the excited electron, the prototypical [Ru(bpy)_3_]^2+^ should behave in the same way as **RubNCS^+^** and **RubCN^+^**, as the bpy-centered orbitals that feature the excited electron are rather independent of the ancillary ligands and the symmetry around the metal center. However, none of the transient absorption experiments performed by different groups in the references gave rise to the dynamics presented in this contribution. Particularly, Hauser and co-workers explored the excited state dynamics of [Ru(bpy)_3_]^2+^ with femtosecond transient absorption spectroscopy in the visible^63^ and near-infrared^64^ regions upon photoexcitation at 400 nm (3.1 eV). No dynamics other than the GS recovery in the hundreds of nanoseconds timescale are, however, noted after 20 ps. Additionally, Shaw and Papanikolas reported the monoexponential behavior of [Ru(bpy)_3_]^2+^ with a 10–12 ps lifetime upon 475 nm (2.6 eV) photoexcitation,^65^ consistent with the observations by Hammarström and co-workers upon 480 nm excitation^47^ on the sub-nanosecond timescale.

We strongly believe that the difference in configuration arises from the hole occupation, which could involve one of the several accessible Ru-centered orbitals of similar energy.^66^ In the following section, we use the electronic structural model for the emitting localized MLCT excited states of ruthenium and osmium polypyridines developed by Kober and Meyer.^22^ This model is based upon the results of, for example, Crosby *et al.* ^67,68^ In the tris-, bis- and mono-bpy complexes of Ru(ii), four closely-spaced MLCT states exist at very low energies. Three of them are positioned within 200 cm^–1^ of each other, while the fourth one is at least 800 cm^–1^ higher in energy. A pseudo *C*_2v_ symmetry is considered, in which the *z* axis is the *C*_2_ axis of the bpy radical anion in the excited state (Fig. 1). For the tris-, bis- and mono-bpy complexes, in each case the four MLCT states originate from the same spatial electronic configuration. This configuration evolves, by means of spin symmetry, in four states that transform into four different symmetries. The highest lying state, namely “the fourth MLCT”, features more singlet character than any of the other states. Importantly, several temperature-dependent experiments support this notion and show that the fourth MLCT is thermally populated from the equilibrated MLCT.^69,70^ For example, its enhanced singlet spin character accelerates its decay to the ground state and, in turn, depopulates the MLCT manifold.^71,72^ We believe that the aforementioned behaviour is not responsible for our observation, as the emitting MLCT lifetimes lack any appreciable changes when changing the excitation energy. Additionally, common to all four states is their similar configuration. As such, it would be difficult to explain the very different spectroscopy. The exact nature of the high-energy MLCT state is, nevertheless, unknown to us. In fact, as this state has a strongly mixed spin and orbital character, we cannot rule out that our high energy state is the fourth MLCT.

We hypothesize that the discrepancies between the emissive states and ES1_505 nm_ might be due to the different spatial orientation of the orbital containing the hole. For example, a consequence of a different hole configuration in the intermediate and in the emissive MLCT might be related to the interactions with the X ligand. In one of these MLCT states, the hole might sit in a d orbital with the symmetry required to interact with the X ligand. As a result, in that state the hole might be extended over the {Ru–X} moiety. This could give rise to different spectroscopy for this state, in comparison with a conventional MLCT state, where the hole sits in an orbital of a different symmetry and is therefore closer to a pure metal-based description. This would allow for photoinduced absorptions to mask the bleaching in the case of the emissive states, but not in the case of ES1_505 nm_, and would also account for the observed enhancement of the PIA for **RubNCS^+^** at 600–610 nm. The same argument would also explain the difference between the electrochemical (GS)HOMO and the spectroscopic non-(GS)HOMO orbitals. We postulate that ES1_505 nm_ is a Franck–Condon ^3^MLCT excited state with the hole sitting in a metal-centered orbital different to the (GS)HOMO. ES1_505 nm_ features a sub-10 ps lifetime and a spectral profile that is not markedly different from that of ES2_505 nm_. The interconversion between ES1_505 nm_ and ES2_505 nm_ is associated with the development of the MLCT manifold on a timescale of a few picoseconds as observed in related molecules.^47–51^

In our interpretation, *k*_23_^505 nm^ (Fig. 5) relates to an internal conversion between two ^3^MLCT excited states. It can also be described as a hole reconfiguration. In **RubNCS^+^**, the remarkable enhancement of the dπ(Ru) ← π(NCS) LMCT transition (Fig. 3) would be the direct consequence of the hole moving to the (GS)HOMO. In short, better orbital overlap and an intensified dπ(Ru) ← π(NCS) LMCT transition in the emissive state would evolve. In contrast, the symmetry of the non-(GS)HOMO metal-centered orbital would provide poorer overlap with NCS^–^ in ES2_505 nm_, which renders the corresponding LMCT transition less intense.

*k*_23_^505 nm^ is associated with an activation barrier, whose origin is intriguing. We consider two alternative explanations, which are both based on our interpretation that the internal conversion is a non-(GS)HOMO → (GS)HOMO hole reconfiguration. On one hand, it is well-known that the one-electron oxidation of ruthenium cyanides and thiocyanates has a strong impact on metal–ligand bond distances as well as intra-ligand bond distances. For example, the average Ru–C distance in K_4_[Ru(CN)_6_] increases from 1.912 Å upon oxidation to 2.050 Å in K_3_[Ru(CN)_6_] due to reduced backbonding effects.^73^ Additionally, it has been shown that the N-bound thiocyanate is best described as {N≡C–S} for Ru(ii), but {N=C=S} for Ru(iii).^30^ It is known from spectroelectrochemical near-infrared measurements and theoretical calculations^23,30^ that in ruthenium thiocyanate and ruthenium cyanide complexes the (GS)HOMOs are spread well over such ligand-centered orbitals. Thus, the (GS)HOMO(h^+^)–(GS)LUMO(e^–^) MLCT electronic configurations for **RubNCS^+^** and **RubCN^+^** mimic those of the Ru(iii) species, as shown by spectroelectrochemistry. We hypothesize that, given the different overlap with the X ligand, a non-(GS)HOMO → (GS)HOMO hole reconfiguration is likely to affect the metal-X ligand and intra-X ligand bond distances. An immediate consequence would be the considerable internal reorganization energies and activation barriers. Likewise, Ru–N(imine) distances could also be subject to reorganization, from which activation barriers would evolve. We find it difficult, however, to believe that such a barrier originates from a transition between states of the same spatial electronic symmetry, regardless of their total symmetry. In any case, femtosecond mid-IR experiments would be very valuable in determining the origin of this barrier. Of great importance would be the C–N stretchings of CN^–^ and NCS^–^, as well as the C–C vibrations of the bpy ligand.^74,75^

Our rationale also explains why the hole reconfiguration phenomenon within MLCT manifolds of ruthenium polypyridines has not yet been reported. It is primarily a consequence of the very low symmetry of {Ru(tpm)(bpy)} complexes, which results in a splitting of the dπ orbitals and in ^3^MLCTs with spectral features, depending on the particular hole configuration. The minor density of MLCT states, stemming from the presence of only one bpy in our complexes, enables the rather unusual phenomena. We believe that a similar behavior could be observed in different low-symmetry polypyridinic Ru complexes, such as {Ru(tpy)(bpy)}, *etc.* Notably, femtosecond transient absorption measurements have already been carried out on some of them,^40^ but their lifetimes are usually compromised by MC states precluding observations analogous to those presented here.

## Experimental

**(RubNCS)PF_6_** and **(RubCN)PF_6_** were synthesized as previously reported.^25^ Acetonitrile for spectroelectrochemical measurements was distilled and dried over CaH_2_. UV-visible spectra were recorded with a Hewlett-Packard 8453 diode array spectrometer (range 190–1100 nm). All the spectroelectrochemical (SEC) experiments were performed using a three-electrode OTTLE cell,^76^ with millimolar solutions of the samples using [TBA]PF_6_ 0.1 M as the supporting electrolyte. Ultrafast transient absorption (TA) experiments were conducted using an amplified Ti/sapphire laser system (Clark MXR CPA2101, FWHM = 150 fs, *λ*_exc_ = 387 nm and 505 nm, 200 nJ per pulse) with TA pump/probe EOS and Helios detection systems from Ultrafast Systems. White light was generated using a sapphire crystal. The optical densities (ODs) of the samples were around 0.5 at the excitation wavelengths. Argon-degassed anhydrous DMSO (99.9% from Aldrich) was employed to eliminate oxygen. A magic angle configuration was employed to avoid rotational dynamics and the chirp generated in the broadband probe was corrected with a polynomial fit before data analysis. Global and target analyses were performed using the GloTarAn software and the R package TIMP.^77^ Further details are given in the ESI.†

## Conclusions

The picosecond excited state dynamics of **RubNCS^+^** and **RubCN^+^** have been characterized through the powerful combination of transient absorption measurements and spectroelectrochemistry. Their lowest triplet excited state is the emissive ^3^MLCT excited state, which is reached as a final reservoir following a cascade of excited state deactivations that start either upon 387 nm or 505 nm illumination. The differential absorption spectroscopy of these states can be accurately reproduced by superimposing those of the one-electron oxidized and one-electron reduced forms. As such, their electronic configuration corresponds to a (GS)HOMO(h^+^)–LUMO(e^–^) charge transfer state.

While photoexcitation at 387 nm results in, after a few picoseconds, an excited state with a (GS)HOMO(h^+^)–(GS)LUMO(e^–^) configuration, 505 nm photoexcitation allows for the observation of an intermediate ^3^MLCT state, in which the hole sits in a metal-centered orbital of different symmetry. The disparity between the electronic configuration of the hole in the intermediate and the emissive ^3^MLCTs has two important consequences. On one hand, both states feature very different fingerprint absorptions in transient absorption measurements. On the other hand, the reconfiguration is impeded by a kinetic barrier. As such, the conversion is followed spectroscopically and kinetically on the 300 ps timescale.

Our rare findings suggest that it is possible to take advantage of higher energy ^3^MLCT states prior to their conversion to low-energy triplets. The corresponding intermediate is a stronger oxidant than the emissive ^3^MLCT and thus is promising for oxidative catalysis. As such, it is intriguing to explore systems with similar low symmetry to determine the exact parameters that allow the trapping of the higher energy ^3^MLCT states. In this direction, we are currently exploring the design of coordination compounds, which would enable the utilization of the energy of the intermediate ^3^MLCT states prior to their dissipation.

## Conflicts of interest

There are no conflicts to declare.
