# Supplementary material for: Trapping intermediate MLCT states in low-symmetry {Ru(bpy)} complexes
Source: Chem Sci. 2017 Aug 29;8(11):7434–42. doi: 10.1039/c7sc02670f (PMC5674176; doi:10.1039/c7sc02670f)
Supplement: Supplementary file 1 [file SC-008-C7SC02670F-s001.pdf]

Electronic Supplementary Material (ESI) for Chemical Science.
This journal is © The Royal Society of Chemistry 2017

SUPPLEMENTARY INFORMATION

for

Trapping intermediate MLCT states in low-symmetry {Ru(bpy)} complexes

by

Alejandro Cadranel, Paola S. Oviedo, German E. Pieslinger, Shiori Yamazaki, Valeria D. Kleiman, Luis M. Baraldo, Dirk M. Guldi

Contents	Page
**Electrochemistry**	**S2**
Figure S1	S2
**Femtosecond Transient Absorption Data**	**S3**
Figure S2	S3
Figure S3	S4
**Analysis of Transient Absorption Data**	**S5**
Figure S4	S6
Figure S5	S7
**Nanosecond Transient Absorption Data**	**S8**
Figure S6	S8
Figure S7	S9
Figure S8	S10
Figure S9	S11
Table S1	S11
Figure S10	S12
**References**	**S13**

Electrochemistry

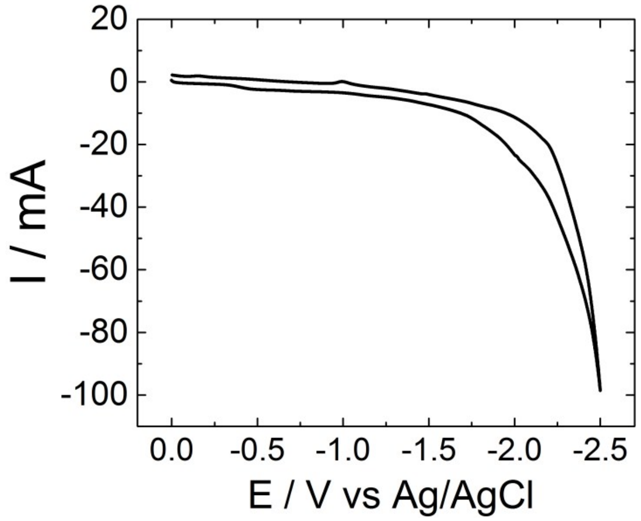

Figure S1. Cyclic voltammetry of a saturated solution of tpm ligand in acetonitrile (0.1 M [TBA]PF_6). The experiment was parformed using a standard three electrode arrangement consisting of a glassy carbon disc (area = 9.4 mm^2) as the working electrode, a platinum wire as the counter electrode and a silver wire as reference electrode plus an internal ferrocene (Fc) standard.

Femtosecond Transient Absorption Data

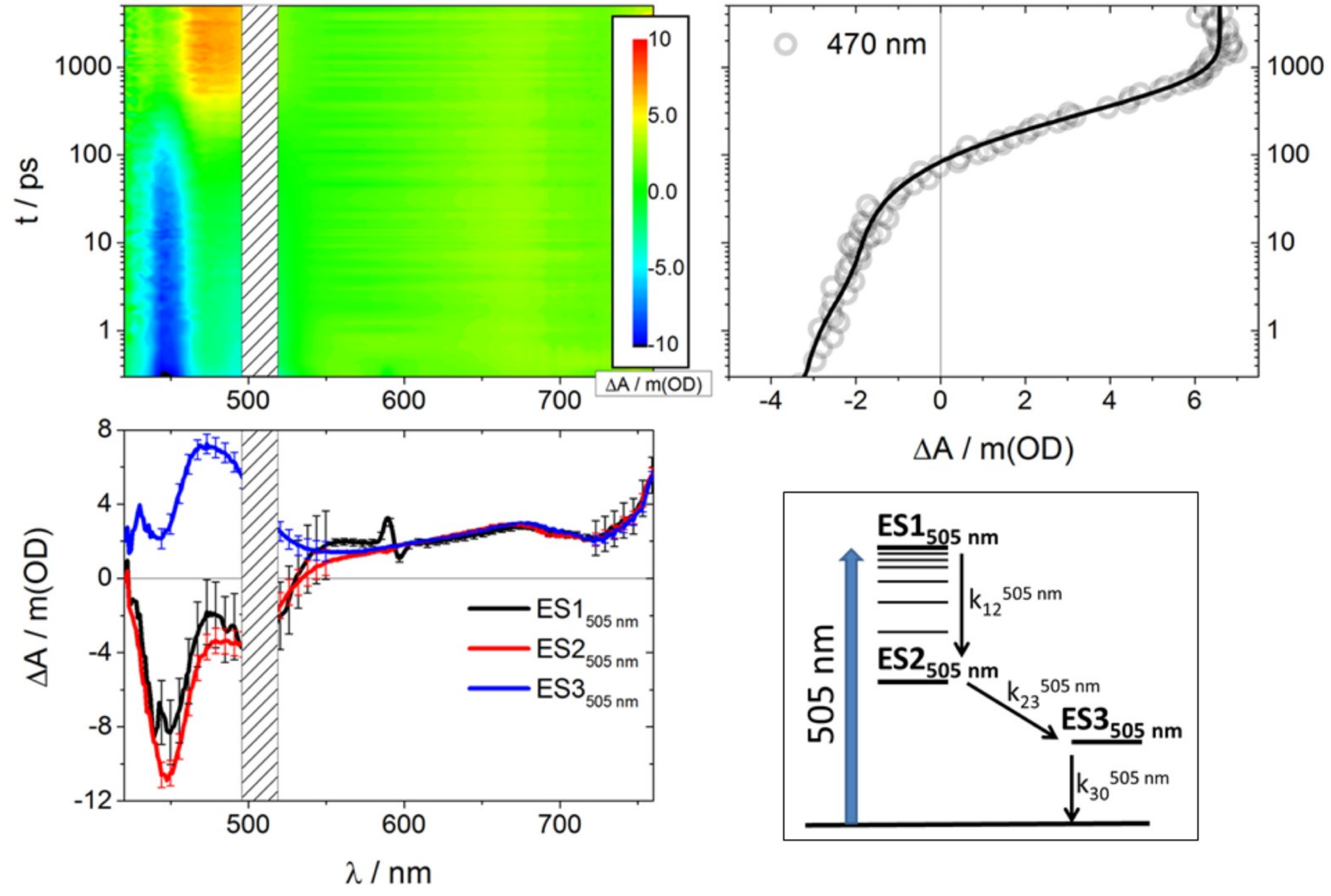

Figure S2. Upper left: Differential absorption 3D map obtained upon femtosecond pump-probe experiments (λ_{ex} = 505 nm) of **RubCN**$^+$ in DMSO at room temperature. Upper right: Time absorption profiles (open circles) and corresponding fittings from Target Analysis (solid lines) for **RubCN**$^+$ upon excitation at 505 nm using the model presented at the bottom right side of this figure. Bottom left: Species associated differential spectra for **RubCN**$^+$ upon excitation at 505 nm – $ES1_{505nm}$: black, $ES2_{505nm}$: red, $ES3_{505nm}$: blue. Bottom right: Target model proposed to fit the data.

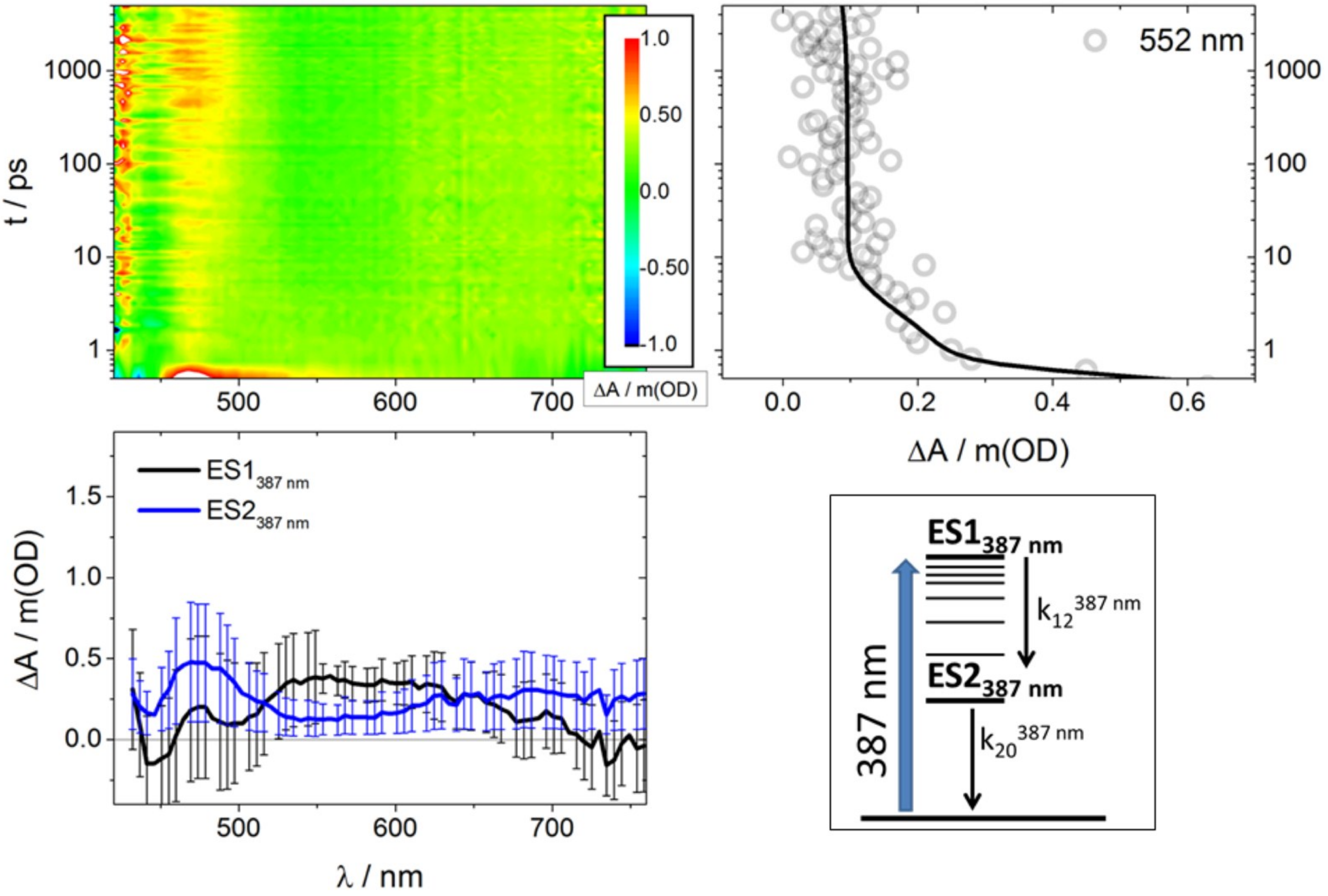

Figure S3. Upper left: Differential absorption 3D map obtained upon femtosecond pump-probe experiments (λ_{ex} = 387 nm) of **RubCN^{+}** in DMSO at room temperature. Upper right: Time absorption profiles (open circles) and corresponding fittings from Target Analysis (solid lines) for **RubCN^{+}** upon excitation at 387 nm using the model presented at the bottom right side of this figure. Bottom left: Species associated differential spectra for **RubCN^{+}** upon excitation at 387 nm – $ES1_{387nm}$: black, $ES2_{387nm}$: red, $ES3_{387nm}$: blue. Bottom right: Target model proposed to fit the data.

Analysis of Femtosecond Transient Absorption Data

Transient absorbance data was analyzed in two steps using GloTarAn Software.[1–4] In first place, survey Global fits were done to estimate the number of spectral components in the transient absorption data. As suggested,[2] a sequential model was used, increasing the number of compartments until the Singular Value Decomposition analysis of the residual matrix gave no indication of additional components. The instrument response function (IRF) was estimated to be ca. 250 fs from the width of the coherent artifacts. The global fits were carried out by convolution of a Gaussian IRF with a multiexponential model.

For 505 nm illumination, four components were needed to fit experimental data (five for **RubNCS$^+$**). Again, the first (and second) one was discarded for similar reasons. The second, third and fourth (third, fourth and fifth) components were taken into account to propose target models, as discussed in the manuscript. Decay associated difference spectra (DADS) resulting from global analysis are shown in Figure S4. The associated time constants were used as initial guess for the target fit.

In the case of 387 nm excitation, three components were needed to fit experimental data. The first component presents a meaninglessly short lifetime, given the resolution of our experiment, and also a spiky shape. The cross phase modulation signals near time zero caused this effect, requiring one component with unphysical spectra and time constants to fit the coherent artifacts.[5] Second and third components were physically meaningful, so these were taken into account to propose a model and run a target analysis. Decay associated difference spectra (DADS) resulting from global analysis are shown in Figure S5. The associated time constants were used as initial guess for the target fit.

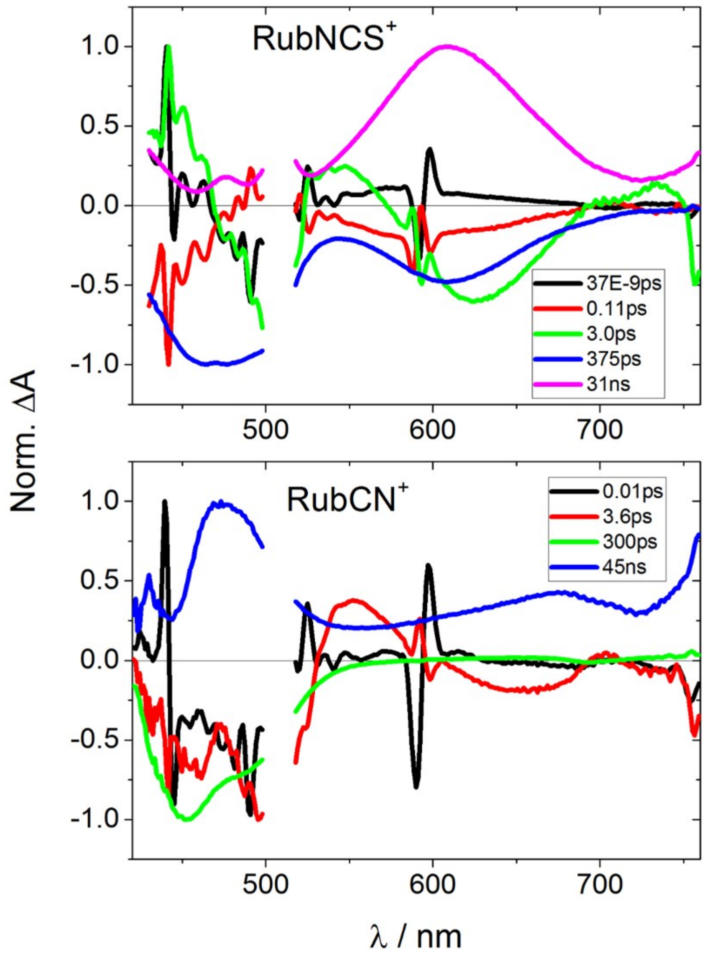

Figure S4. Decay associated difference spectra (DADS) for **RubNCS⁺** (top) and **RubCN⁺** (bottom) in DMSO at room temperature (λ pump: 505 nm). See text on page S5 for details.

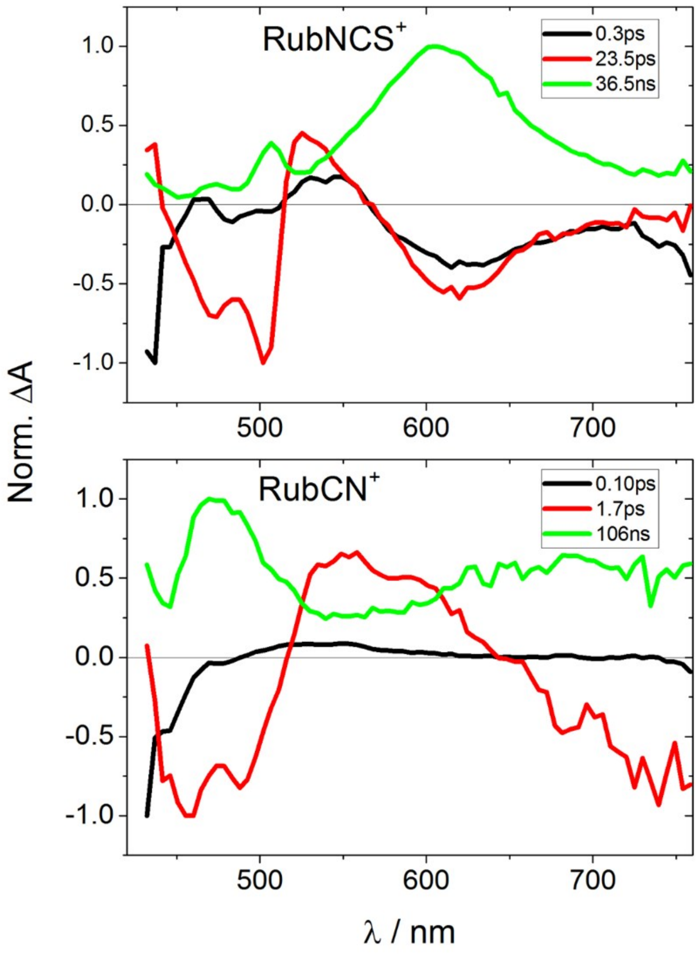

Figure S5. Decay associated difference spectra (DADS) for **RubNCS⁺** (top) and **RubCN⁺** (bottom) in DMSO at room temperature (λ pump: 387 nm). See text on page S5 for details.

.

Nanosecond transient absorption spectroscopy

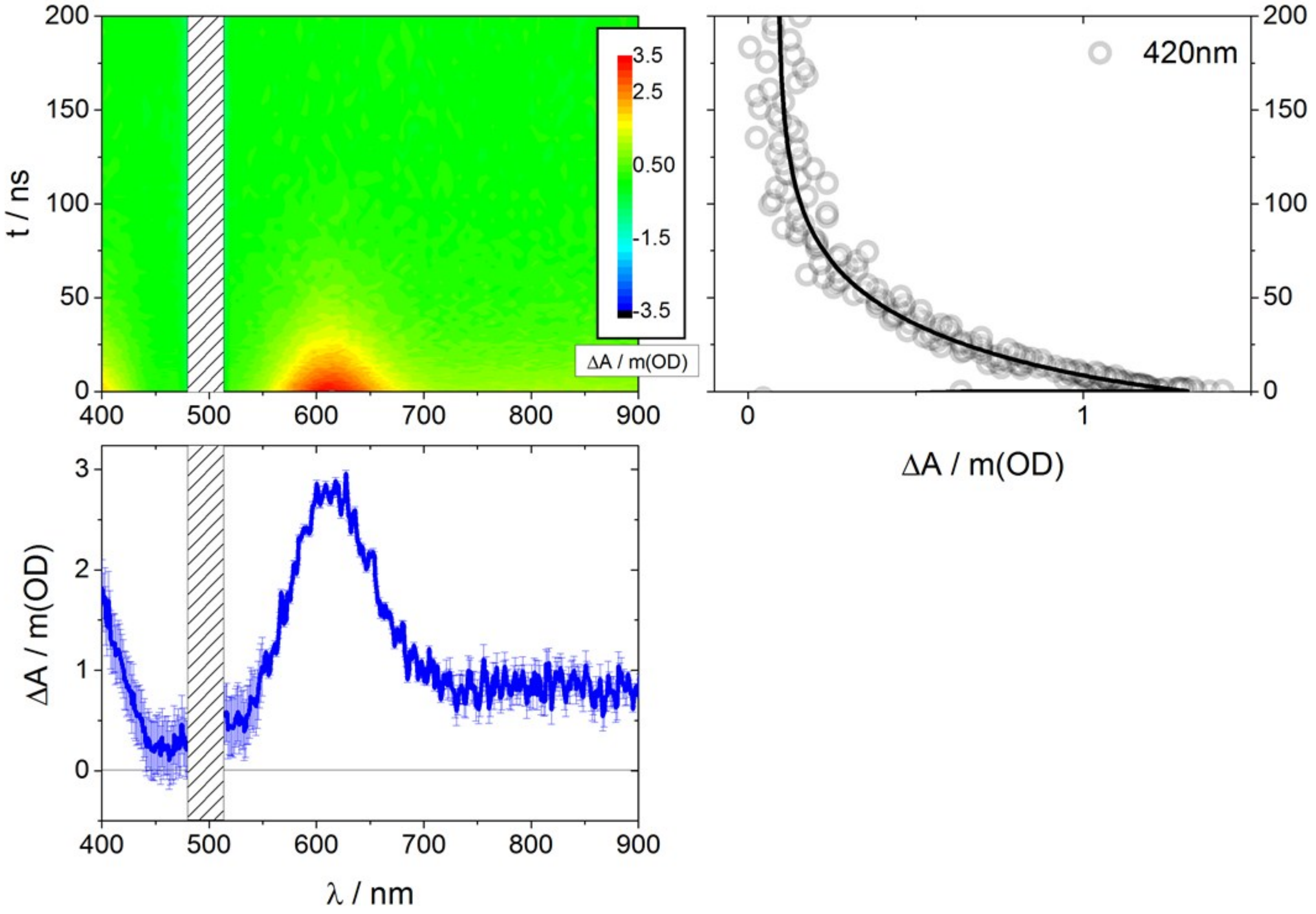

Figure S6. Upper left: Differential absorption 3D map obtained upon nanosecond pump-probe experiments (λ_{ex} = 505 nm) of **RubNCS⁺** in DMSO at room temperature. Upper right: Time absorption profiles (open circles) and corresponding monoexponential fittings (solid lines) for **RubNCS⁺** upon excitation at 505 nm. Bottom left: Species associated differential spectra for **RubNCS⁺** upon excitation at 505 nm.

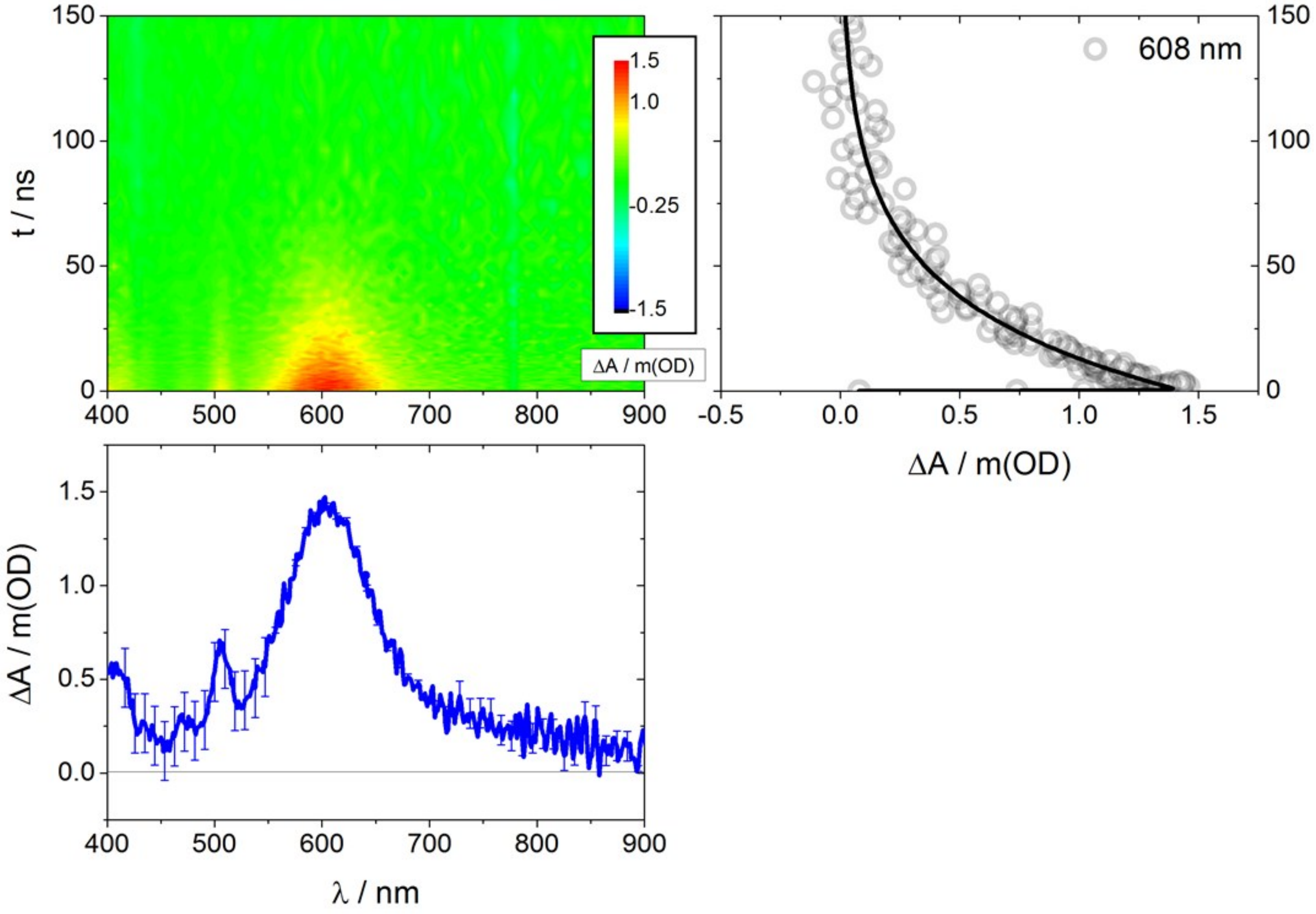

Figure S7. Upper left: Differential absorption 3D map obtained upon nanosecond pump-probe experiments (λ_{ex} = 387 nm) of **RubNCS**$^+$ in DMSO at room temperature. Upper right: Time absorption profiles (open circles) and corresponding monoexponential fittings (solid lines) for **RubNCS**$^+$ upon excitation at 387 nm. Bottom left: Species associated differential spectra for **RubNCS**$^+$ upon excitation at 387 nm.

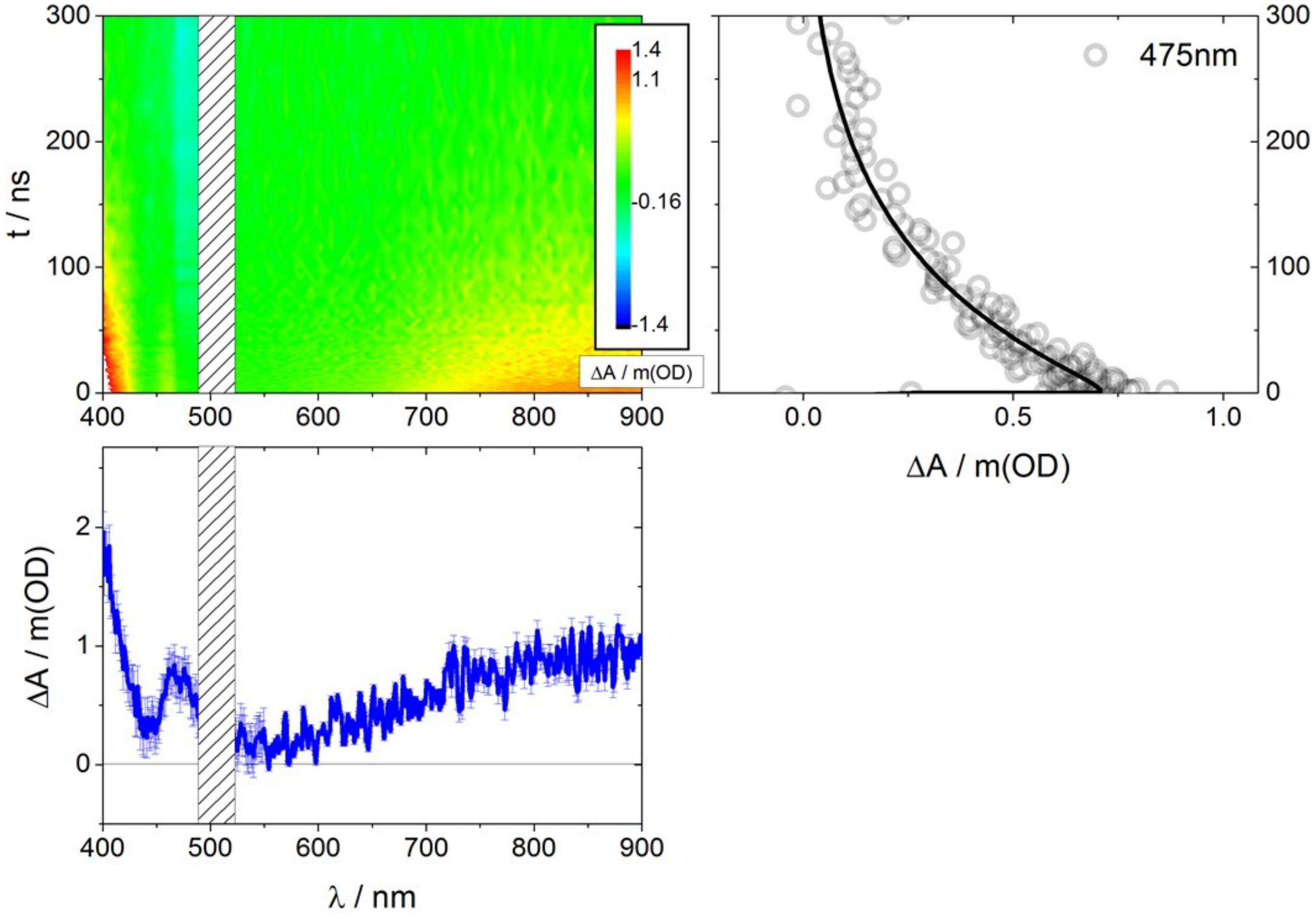

Figure S8. Upper left: Differential absorption 3D map obtained upon nanosecond pump-probe experiments (λ_{ex} = 505 nm) of **RubCN**$^+$ in DMSO at room temperature. Upper right: Time absorption profiles (open circles) and corresponding monoexponential fittings (solid lines) for **RubCN**$^+$ upon excitation at 505 nm. Bottom left: Species associated differential spectra for **RubCN**$^+$ upon excitation at 505 nm.

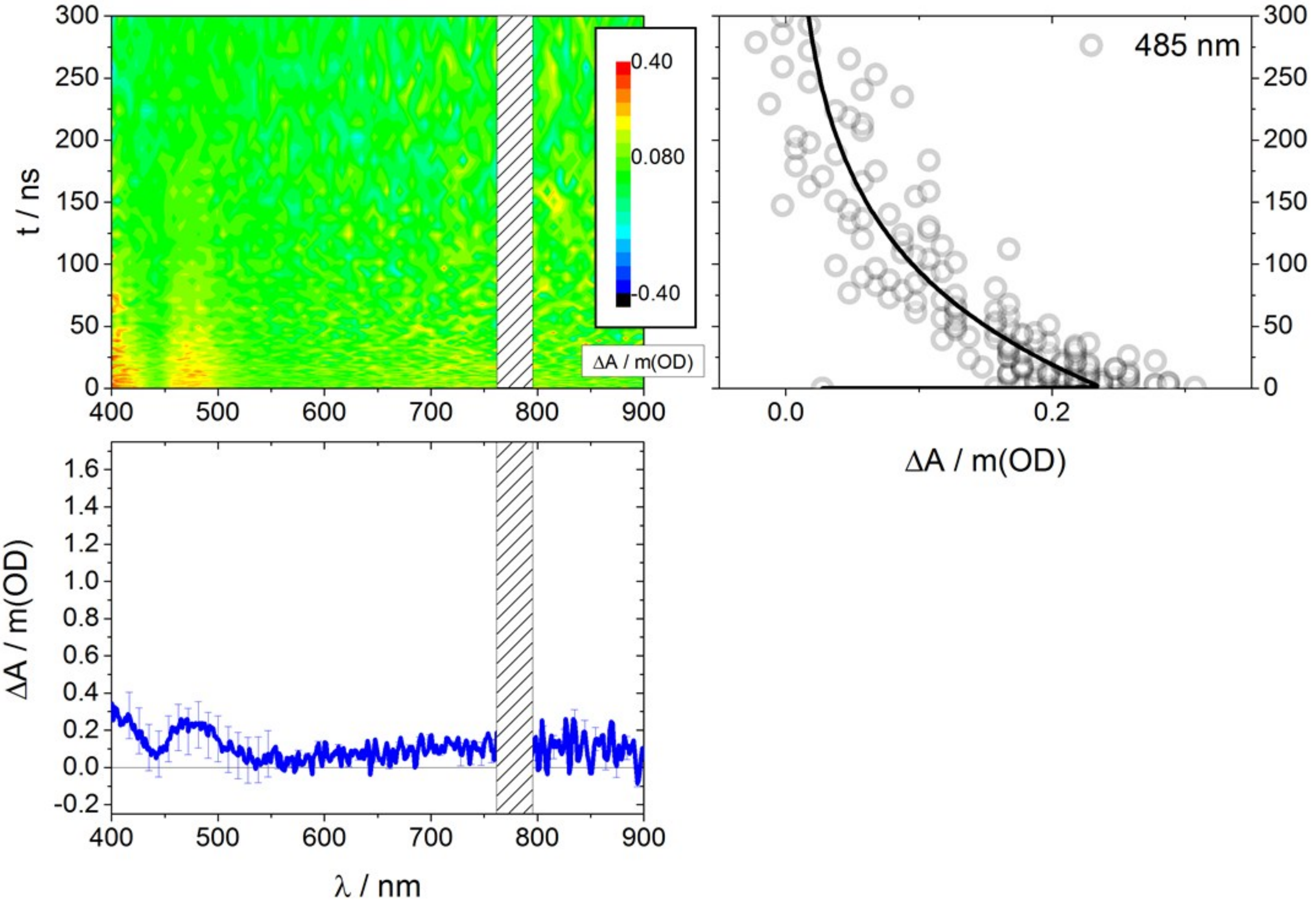

Figure S9. Upper left: Differential absorption 3D map obtained upon nanosecond pump-probe experiments (λ_{ex} = 387 nm) of **RubCN⁺** in DMSO at room temperature. Upper right: Time absorption profiles (open circles) and corresponding monoexponential fittings (solid lines) for **RubCN⁺** upon excitation at 387 nm. Bottom left: Species associated differential spectra for **RubCN⁺** upon excitation at 387 nm.

	λ_{pump} = 387 nm	λ_{pump} = 505 nm
	k_{20} / μs^{-1} (τ_{20} / ns)	k_{20} / μs^{-1} (τ_{20} / ns)
**RubNCS⁺**	27.88 ± 0.06 (35.9)	28.27 ± 0.07 (35.4)
**RubCN⁺**	9.4 ± 0.4 (106)	8.89 ± 0.06 (112.5)

Table S1. Time constants for ground state recovery in photoexcited **RubNCS⁺** and **RubCN⁺** in DMSO at room temperature.

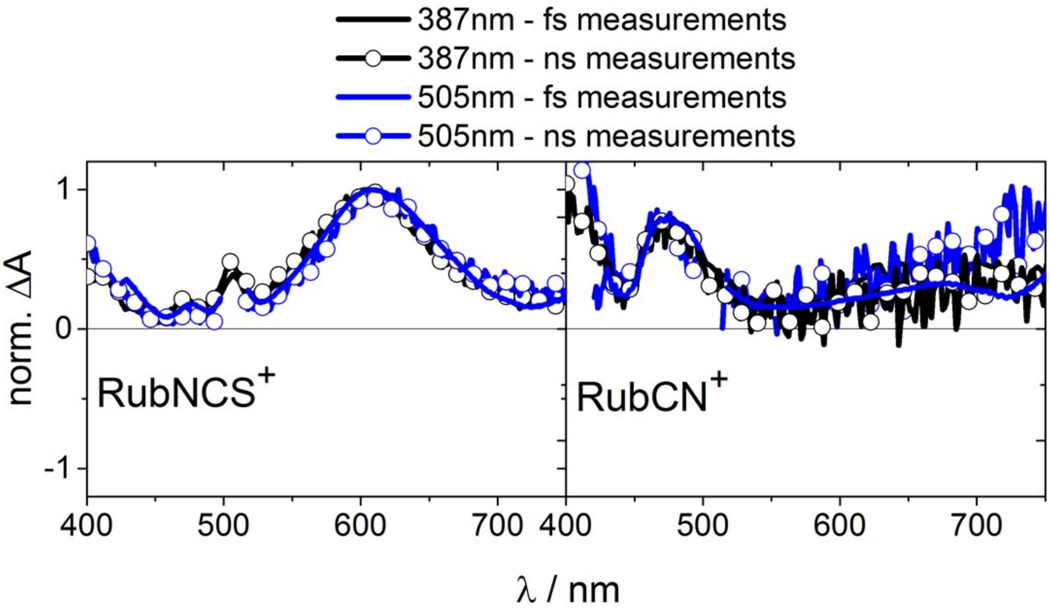

Figure S10. Comparison between the spectra for $ES3_{387nm}$ (black curves) and $ES3_{505nm}$ (blue curves), obtined by femtosecond pump-probe spectroscopy, and the monoexponentially deyaing spectra upon 387 nm excitation (black curve with circles) and 505 nm (blue curve with circles) obtained by nanosecond pump-probe spectroscopy, for **RubNCS$^+$** and **RubCN$^+$** in DMSO at room temperature

References

(1) van Wilderen, L. J. G. W.; Lincoln, C. N.; van Thor, J. J. Modelling Multi-Pulse Population Dynamics from Ultrafast Spectroscopy. *PLoS One* **2011**, *6* (3), e17373 DOI: 10.1371/journal.pone.0017373.

(2) Van Stokkum, I. H. M.; Larsen, D. S.; Van Grondelle, R. Global and Target Analysis of Time-Resolved Spectra. *Biochim. Biophys. Acta - Bioenerg.* **2004**, *1657* (2–3), 82–104 DOI: 10.1016/j.bbabio.2004.04.011.

(3) Snellenburg, J. J. Glotaran - a Tool for Interactive Global and Target Analysis of Time-Resolved Spectroscopy and Microscopy Data. **2010**, 70.

(4) Snellenburg, J. J.; Laptenok, S. P.; Seger, R.; Mullen, K. M.; van Stokkum, I. H. M. Glotaran : A Java -Based Graphical User Interface for the R Package TIMP. *J. Stat. Softw.* **2012**, *49* (3) DOI: 10.18637/jss.v049.i03.

(5) Foing, J.-P.; Joffre, M.; Oudar, J.-L.; Hulin, D. Coherence Effects in Pump-Probe Experiments with Chirped Pump Pulses. *J. Opt. Soc. Am. B* **1993**, *10* (7), 1143–1148 DOI: 10.1364/JOSAB.10.001143.
